# An Operator Analysis on Stochastic Differential Equation (SDE)-Based Diffusion Generative Models

**DOI:** 10.3390/e28030290

**Published:** 2026-03-04

**Authors:** Yunpei Wu, Yoshinobu Kawahara

**Affiliations:** 1 Faculty of Mathematics, Kyushu University, Fukuoka 819-0395, Japan; 2 Graduate School of Information Science and Technology, The University of Osaka, Osaka 565-0871, Japan; kawahara@ist.osaka-u.ac.jp

**Keywords:** generative modeling, SDE, eigenfunction decomposition, Fokker–Planck operator, kernel methods

## Abstract

Score-based generative models, grounded in stochastic differential equations (SDEs), excel in producing high-quality data but suffer from slow sampling due to the extensive nonlinear computations required for iterative score function evaluations. We propose an innovative approach that integrates score-based reverse SDEs with kernel methods, leveraging the derivative reproducing property of reproducing kernel Hilbert spaces (RKHSs) to efficiently approximate the eigenfunctions and eigenvalues of the Fokker–Planck operator. This enables data generation through linear combinations of eigenfunctions, transforming computationally intensive nonlinear operations into efficient linear ones, thereby significantly reducing computational overhead. Notably, our experimental results demonstrate remarkable progress: despite a slight reduction in sample diversity, the sampling time for a single image on the CIFAR-10 dataset is reduced to an impressive 0.29 s, marking a substantial advancement in efficiency. This work introduces novel theoretical and practical tools for generative modeling, establishing a robust foundation for real-time applications.

## 1. Introduction

Generative modeling is a central theme in modern machine learning, enabling applications in image synthesis, audio generation, and scientific data simulation [1]. Among existing approaches, diffusion/score-based generative models have become particularly successful due to their strong empirical sample quality and principled training objectives [2,3]. In the continuous-time formulation of Song et al. [4], a forward SDE gradually perturbs data into noise, and sampling is performed by simulating a reverse-time SDE whose drift depends on the score $\nabla_{x}\log p_{t}\left(x\right)$, approximated by a neural network. While this framework (including VE/VP SDEs) achieves state-of-the-art results on datasets such as CIFAR-10 [4], its practical limitation is well known: sampling typically requires hundreds to thousands of score-network evaluations, leading to seconds per image and limiting real-time or on-device usage.

A broad line of work accelerates diffusion sampling by reducing discretization steps (e.g., DDIM [5]), improving numerical solvers (e.g., DPM-Solver [6]), moving generation to a latent space [7], reformulating the generative dynamics as Flow Matching [8], or distilling the model into fewer steps [9,10]. These approaches are effective, but they still produce samples by iterating a time-stepping procedure whose dominant cost is repeated nonlinear evaluations of a learned model, and they may require careful tuning to balance speed and quality.

This paper explores an alternative route: instead of accelerating the numerical integration itself, we approximate the linear operator that governs the evolution of the probability density along the reverse-time dynamics. Concretely, given a trained score network, the reverse-time SDE defines a time-inhomogeneous diffusion process, and the associated Fokker–Planck operator $L^{*}$ describes how the density $p_{t}\left(x\right)$ evolves. Operator theory suggests that, when a useful low-dimensional spectral representation exists, the density evolution can be approximated by a small number of eigenmodes of $L^{*}$ [11]. Our key idea is to approximate such eigenmodes in a reproducing kernel Hilbert space (RKHS) using kernel-based generator methods [12] and then use the resulting spectral expansion to construct an explicit approximation of the terminal density at t=0. This operator-based strategy introduces a different computational trade-off. The spectral approximation requires an offline eigendecomposition of kernel Gram matrices, whose cost grows cubically with the number of reference data points. However, this step is performed only once. During computation, the method replaces repeated nonlinear neural network evaluations with linear combinations of a small number of eigenfunctions, leading to substantial acceleration. The trade-off is that the data density is approximated by a truncated spectral expansion, which may introduce approximation bias and reduce sample diversity compared to score-based reverse SDE generation.

At a high level, the method proceeds as follows. We (i) use kernel-based approximations of infinitesimal generators to estimate a finite-dimensional eigendecomposition of the Fokker–Planck operator $L^{*}$ in an RKHS; (ii) represent the density by a truncated eigenfunction expansion, yielding an explicit approximation ${\hat{p}}_{0}\left(x\right)$ of the data density at time t=0; and (iii) generate samples using only the corrector component of predictor–corrector sampling by running Langevin dynamics driven by the closed-form score $\nabla_{x}\log{\hat{p}}_{0}\left(x\right)$. Importantly, the score $\nabla_{x}\log{\hat{p}}_{0}\left(x\right)$ can be evaluated using kernel and eigenfunction evaluations, avoiding repeated calls to the original neural score model during sampling.

This viewpoint makes the computational trade-off transparent. A standard PC sampler with *N* time steps performs O(N) evaluations of a large neural network per sample (and typically uses $N\approx 10^{2}$–$10^{3}$), which dominates runtime. In contrast, our sampler replaces these nonlinear evaluations with O(NLM) kernel-based computations, where *M* is the number of reference points used in the RKHS representation and *L* is the number of retained eigenmodes. Since kernel/eigenfunction evaluations are lightweight compared to a U-Net-scale score network, this substitution can yield large speedups in wall-clock time. Empirically, we observe 100–260× faster single-image sampling on CIFAR-10 compared with PC1000 baselines, while maintaining recognizable sample structure, albeit with a noticeable degradation in diversity as measured by FID/IS. These results suggest that spectral operator approximations can substantially reduce sampling cost, and they motivate future work on improving the spectral density approximation to narrow the quality gap.

In summary, the contributions of this work are:

- Operator-based sampling formulation for score-based SDEs. We connect reverse-time diffusion sampling to the spectral structure of the associated Fokker–Planck operator $L^{*}$ and propose to approximate its leading eigenmodes in an RKHS.
- Kernel eigenfunction density representation. We derive a practical truncated eigenfunction expansion that yields an explicit density approximation ${\hat{p}}_{0}\left(x\right)$ and a closed-form score $\nabla_{x}\log{\hat{p}}_{0}\left(x\right)$ computable via kernel derivatives.
- Fast sampling via corrector-only dynamics. Using the above score, we implement a corrector-only Langevin sampler that avoids repeated neural score evaluations at sampling time and achieves large empirical speedups on CIFAR-10.

This paper is organized as follows: Section 2 surveys related work, Section 3 describes the proposed operator-based method, Section 4 reports experimental results, and Section 5 concludes.

## 2. Related Work

### 2.1. Score-Based SDE Models

Score-based generative models, unified by Song et al. [4], model data generation as a continuous diffusion process, generalizing DDPM [13] and SMLD [14]. A forward SDE perturbs data into noise over t∈[0,T]:

(1) dx=f(x,t)dt+g(t)dw,

where $f\left(x,t\right)={\left[f_{1},f_{2},\ldots ,f_{d}\right]}^{⊤}$ is the drift, g(t) the diffusion coefficient, and w a Wiener process. The reverse SDE, derived from the forward SDE via time reversal [15], runs from t=T to t=0 to generate samples:

(2) $$dx=\left[f\left(x,t\right)-g{\left(t\right)}^{2}\nabla_{x}\log p_{t}\left(x\right)\right]dt+g\left(t\right)d\bar{w},$$

where $p_{t}$ denotes the probability density function at time *t*, $\nabla_{x}\log p_{t}\left(x\right)$ is the score function and $\bar{w}$ is a reverse-time Wiener process. Since the score $\nabla_{x}\log p_{t}\left(x\right)$ is unknown, it is approximated by a neural network $s_{\theta }\left(x,t\right)$:

(3) $$\theta^{*}=\arg\min_{\theta }E_{t}\left\{\lambda \left(t\right)E_{x(0)}E_{x(t)|x(0)}\left[{∥s_{\theta }\left(x\left(t\right),t\right)-\nabla_{x(t)}\log p_{0t}\left(x\left(t\right)|x\left(0\right)\right)∥}_{2}^{2}\right]\right\},$$

where λ(t) weights the loss, often set as $\lambda \left(t\right)\propto 1/E\left[{∥\nabla_{x(t)}\log p_{0t}\left(x\left(t\right)|x\left(0\right)\right)∥}_{2}^{2}\right]$. Sampling discretizes the reverse SDE using predictor–corrector (PC) methods, iterating for i=N,…,1,

(4) $$x_{i-1}=x_{i}+\left[f\left(x_{i},t_{i}\right)-g{\left(t_{i}\right)}^{2}s_{\theta }\left(x_{i},t_{i}\right)\right]\Delta t+g\left(t_{i}\right)\sqrt{\Delta t}z_{i},$$

followed by Langevin MCMC corrections, where $z_{i}∼N\left(0,I\right)$. Alternatively, a probability flow ODE,

(5) $$dx=\left[f\left(x,t\right)-\frac{1}{2}g{\left(t\right)}^{2}s_{\theta }\left(x,t\right)\right]dt,$$

enables deterministic sampling. Variants include the variance-exploding (VE) SDE with growing variance and variance-preserving (VP) SDE with fixed variance [16].

### 2.2. Kernel-Based Approximation of the Koopman Generator

Kernel-based methods, as proposed by Klus et al. [12], enable efficient approximation of the Koopman generator’s eigendecomposition for stochastic dynamical systems. For a system governed by SDE (1), the Koopman generator L acts on an observable function $h:R^{d}\to R$ as follows:

(6) $$Lh=\sum_{i=1}^{d}f_{i}\left(x,t\right)\frac{\partial h}{\partial x_{i}}+\frac{1}{2}\sum_{i=1}^{d}\sum_{j=1}^{d}g{\left(t\right)}^{2}\frac{\partial^{2}h}{\partial x_{i}\partial x_{j}},$$

The eigendecomposition of the Koopman generator, given by $L\varphi_{\ell }=\lambda_{\ell }\varphi_{\ell }$, yields eigenvalues $\lambda_{\ell }$ and eigenfunctions $\varphi_{\ell }$, capturing the system’s long-term dynamics through its spectral properties.

**Definition** **1.** 
*Let $R^{d}$ be the state space and H a space of functions $f:R^{d}\to R$. Then, H is a reproducing kernel Hilbert space (RKHS) with inner product ${\langle \cdot ,\cdot \rangle }_{H}$ if a kernel $k:R^{d}\times R^{d}\to R$ exists such that:*

*(i)*  *${\langle f,k\left(x,\cdot \right)\rangle }_{H}=f\left(x\right)$ for all f∈H and $x\in R^{d}$;*
*(ii)*  *$H=\bar{\mathrm{span}}\left\{k\left(x,\cdot \right)|x\in R^{d}\right\}$.*

The RKHS, defined by a kernel $k(x,x^{\prime })$, uses the feature mapping ϕ(x)=k(x,·) to approximate L’s eigendecomposition, where $k\left(x_{m},x_{r}\right)={\langle \phi \left(x_{m}\right),\phi \left(x_{r}\right)\rangle }_{H}$. Given data ${\left\{x_{m}\right\}}_{m=1}^{M}∼p_{t}\left(x\right)$, let $\phi_{m}\left(\cdot \right)=k\left(x_{m},\cdot \right)$. Gram matrices are constructed:

(7) $${\left[G_{0}\right]}_{mr}={\langle \phi_{m},\phi_{r}\rangle }_{H},\;{\left[G_{2}\right]}_{mr}={\langle L\phi_{m},\phi_{r}\rangle }_{H},$$

where ${\langle L\phi_{m},\phi_{r}\rangle }_{H}$ applies (6) to $\phi_{m}$ in the RKHS inner product. Solving the generalized eigenvalue problem $G_{2}u_{\ell }=\lambda_{\ell }G_{0}u_{\ell }$ yields approximations ${\hat{\lambda }}_{\ell }$ and ${\hat{\varphi }}_{\ell }\left(x\right)=\sum_{m=1}^{M}u_{\ell ,m}\phi_{m}\left(x\right)$. With sufficient data, ${\hat{\varphi }}_{\ell }\to \varphi_{\ell }$ [12].

Score-based SDE models in Section 2.1 achieve strong expressive power by learning nonlinear score functions and have demonstrated state-of-the-art sample quality. However, their sampling procedure requires repeated evaluations of the neural network across many time steps, resulting in a substantial computational cost.

Kernel-based generator methods in Section 2.2, in contrast, provide a linear operator perspective on stochastic dynamics. Through spectral decomposition of the associated generator, density evolution can be represented in terms of eigenfunctions. This representation offers structural interpretability.

The proposed method is motivated by combining these two viewpoints. We retain the learned score-based reverse SDE to define the underlying stochastic dynamics, while approximating the associated Fokker–Planck operator in an RKHS to obtain a truncated spectral representation. This enables sampling through linear combinations of eigenfunctions, thereby reducing sampling complexity compared to direct reverse-time SDE simulation.

## 3. Proposed Method

### 3.1. Fokker–Planck Operator Eigendecomposition

For an Itô SDE, the time evolution of the probability density is governed by the Fokker–Planck operator, which is the adjoint of the Koopman generator associated with the underlying stochastic dynamics. While the Koopman generator L acts on observables, its adjoint $L^{*}$ acts on densities and characterizes how probability mass evolves in time.

**Definition 2** 
(Fokker–Planck Operator)**.** Let L denote the Koopman generator of the forward SDE (1). The Fokker–Planck operator $L^{*}$ is defined as the adjoint of L with respect to the standard $L^{2}\left(R^{d}\right)$ inner product, i.e.,

$$\langle Lh,j\rangle =\langle h,L^{*}j\rangle ,\;\forall h,j\in C_{0}^{\infty }\left(R^{d}\right),$$

*and is given explicitly by*

(8) $$L^{*}p=-\sum_{i=1}^{d}\frac{\partial }{\partial x_{i}}\;\left[f_{i}\left(x,t\right)\;p\left(x\right)\right]+\frac{1}{2}\sum_{i,j=1}^{d}\frac{\partial^{2}}{\partial x_{i}\partial x_{j}}\;\left[g{\left(t\right)}^{2}\;p\left(x\right)\right].$$

*The associated Fokker–Planck equation reads*

$$\frac{\partial p_{t}\left(x\right)}{\partial t}=L^{*}p_{t}\left(x\right).$$

The spectral properties of $L^{*}$ provide a natural way to describe density evolution. In particular, if $L^{*}\varphi_{\ell }=\lambda_{\ell }\varphi_{\ell }$, each eigenfunction $\varphi_{\ell }$ represents a dynamical mode of the density, with temporal behavior governed by $\lambda_{\ell }$. Rather than working with the infinite-dimensional operator directly, we seek a data-driven finite-dimensional approximation of this spectral structure.

Following kernel-based generator approximation methods [12], we approximate the action of $L^{*}$ in a reproducing kernel Hilbert space (RKHS) H defined in Definition 1. Given samples ${\left\{x_{m}\right\}}_{m=1}^{M}∼p_{t}\left(x\right)$, we first construct the Gram matrices $G_{0}$ and $G_{2}$ associated with the Koopman generator L, as in (7). At the discrete level induced by the kernel basis, the adjoint relationship between L and $L^{*}$ is reflected by the transposed Gram matrices,

(9) $${\left[G_{0}^{⊤}\right]}_{mr}={\left[G_{0}\right]}_{rm},\;{\left[G_{2}^{⊤}\right]}_{mr}={\left[G_{2}\right]}_{rm}.$$

We then solve the generalized eigenvalue problem

(10) $$G_{2}^{⊤}u_{\ell }=\lambda_{\ell }\;G_{0}^{⊤}u_{\ell },$$

which yields a finite-dimensional approximation of the Fokker–Planck spectrum. The corresponding eigenfunctions are represented in the RKHS as

$${\hat{\varphi }}_{\ell }\left(x\right)=\sum_{m=1}^{M}u_{\ell ,m}\;k\left(x_{m},x\right),$$

with ${\hat{\lambda }}_{\ell }$ approximating the true eigenvalues $\lambda_{\ell }$.

### 3.2. Probability Density Estimation

We first train a score-based SDE model $s_{\theta }\left(x,t\right)$, obtained as described in Section 2.1 via (3), to define the reverse SDE capable of generating new data samples. For clarity and without loss of rigor, we denote this reverse SDE as follows:

(11) $$dx=h\left(x,t\right)dt+g\left(t\right)d\bar{w},$$

where $h\left(x,t\right)=f\left(x,t\right)-g{\left(t\right)}^{2}s_{\theta }\left(x,t\right)$ is the drift term, incorporating the score function $s_{\theta }\left(x,t\right)\approx \nabla_{x}\log p_{t}\left(x\right)$, g(t) is the diffusion coefficient, and $\bar{w}$ is a standard Wiener process in reverse time. The evolution of the probability density $p_{t}\left(x\right)$ for this SDE is governed by the Fokker–Planck equation:

(12) $$\frac{\partial p_{t}}{\partial t}=L^{*}p_{t},$$

where $L^{*}$ is the Fokker–Planck operator.

It is important to note that although the drift term h(x,t) in the reverse SDE may be highly nonlinear due to the learned score function, the Fokker–Planck Equation (12) is linear in the density $p_{t}\left(x\right)$. The linear structure exploited in this work, therefore, refers to the density evolution operator $L^{*}$ acting on probability densities.

Using the method in Section 3.1, we obtain the eigenvalues $\lambda_{\ell }$ and eigenfunctions $\varphi_{\ell }\left(x\right)=\sum_{m=1}^{M}u_{\ell ,m}k\left(x_{m},x\right)$ of the Fokker–Planck operator $L^{*}$ in the RKHS H defined by the Gaussian kernel $k\left(x,x^{\prime }\right)=\exp\left(-\frac{∥x-x^{\prime }{∥}^{2}}{2\zeta^{2}}\right)$ with bandwidth ζ.

We approximate the Fokker–Planck operator $L^{*}$ in the finite-dimensional RKHS subspace $H_{N}\subset H$ induced by the data. Solving the corresponding generalized eigenvalue problem yields eigenpairs $\left(\lambda_{\ell },\varphi_{\ell }\right)$, where $\varphi_{\ell }\in H_{N}$ are represented through kernel expansions.

Restricting the evolution to this subspace, the density is approximated by the truncated spectral form,

(13) $$p_{t}\left(x\right)\approx \sum_{\ell =1}^{L}c_{\ell }\left(T\right)\;e^{\lambda_{\ell }\left(t-T\right)}\;\varphi_{\ell }\left(x\right),$$

where $L\leq \dim(H_{N})$ denotes the number of retained modes. The coefficients $c_{\ell }\left(T\right)$ are determined by projecting the terminal density onto the computed eigenfunctions within $H_{N}$ (e.g., via an empirical least-squares fit on the reference data).

**Remark 1** 
(Frozen-time approximation and spectral interpretation)**.** In general, the reverse-time SDE is time-inhomogeneous, and the associated Fokker–Planck operator depends explicitly on time. The exact solution of $\partial_{t}p_{t}=L_{t}^{*}p_{t}$ is given by a time-ordered exponential, which does not admit a global time-independent spectral decomposition.

In this work, we adopt a frozen-time approximation: we fix a reference time $\bar{t}$ and approximate the instantaneous operator $L_{\bar{t}}^{*}$ in a finite-dimensional RKHS subspace. All eigenpairs $\left(\lambda_{\ell },\varphi_{\ell }\right)$ are therefore understood as arising from this discretized frozen-time operator.

To estimate $p_{t}\left(x\right)$, we approximate the initial density $p_{T}\left(x\right)$, typically a known prior distribution (e.g., $N(0,\sigma_{\max}^{2}I)$ for VE SDEs or N(0,I) for VP SDEs). Using data points ${\left\{y_{n}\right\}}_{n=1}^{N}∼p_{T}$, we employ kernel density estimation in the RKHS H to obtain

(14) $${\hat{p}}_{T}\left(x\right)=\frac{1}{N}\sum_{n=1}^{N}k\left(x,y_{n}\right).$$

The initial coefficients $c_{\ell }\left(T\right)$ are computed by projecting ${\hat{p}}_{T}\left(x\right)$ onto the eigenfunctions in the RKHS inner product:

(15) $$c_{\ell }\left(T\right)={\langle {\hat{p}}_{T},\varphi_{\ell }\rangle }_{H}\approx \frac{1}{N}\sum_{n=1}^{N}\varphi_{\ell }\left(y_{n}\right).$$

The density at t=0 is approximated by truncating to the *L* dominant eigenfunctions (those with the largest Re(λℓ)):

(16) $${\hat{p}}_{0}\left(x\right)=\sum_{\ell =1}^{L}c_{\ell }\left(T\right)e^{-\lambda_{\ell }T}\varphi_{\ell }\left(x\right).$$

This density ${\hat{p}}_{0}\left(x\right)$ approximates the data distribution and serves as the basis for fast sampling, as described in the subsequent section. The trained score function $s_{\theta }\left(x,t\right)$, used to define h(x,t) in (11), ensures that the Fokker–Planck operator $L^{*}$ captures the reverse SDE dynamics, enabling precise density estimation.

**Remark 2.** 
*The accuracy of ${\hat{p}}_{0}\left(x\right)$ depends on the kernel bandwidth ζ, the truncation level L, and the number of reference data points M in the RKHS approximation.*

*The parameter L determines the number of retained spectral modes of the approximated Fokker–Planck operator. In practice, L is chosen according to the decay of the computed spectrum: when the eigenvalues exhibit rapid decay, the leading modes capture the dominant structure, and larger L yields diminishing returns at increased computational cost. This follows the classical Galerkin-type spectral truncation principle.*

*The parameter M specifies the dimension of the finite RKHS subspace used to approximate the operator. Increasing M improves the resolution of the kernel-based representation, but also raises the computational complexity of the generalized eigenvalue problem and may introduce conditioning issues in the Gram matrices [12,17].*

*The bandwidth ζ controls the locality of the kernel representation: smaller values enhance resolution but may cause overfitting, whereas larger values produce smoother but potentially biased approximations. In practice, Cross-validation techniques [18], including recent nonparametric kernel flow perspectives [19], can optimize these hyperparameters.*

### 3.3. Sampling

Having estimated the probability density ${\hat{p}}_{0}\left(x\right)$ of the data distribution using the spectral representation in Section 3.2, we now describe a method to generate new samples efficiently. Instead of relying on iterative numerical solvers for the reverse SDE (11), as in traditional predictor–corrector (PC) sampling [4], we leverage the analytical form of ${\hat{p}}_{0}\left(x\right)$ to compute a new score function and apply only the corrector component of the PC sampler. Since we explicitly construct an approximation ${\hat{p}}_{0}\left(x\right)$ of the terminal density, sampling is performed directly from this density using Langevin dynamics, rather than by numerically integrating the reverse-time SDE as in predictor–corrector sampling. In this sense, the predictor step is not required because the generation mechanism is based on direct density approximation instead of time-evolution simulation.

To sample from ${\hat{p}}_{0}\left(x\right)$, we first compute the score, defined as the gradient of the log-probability density $\nabla_{x}\log{\hat{p}}_{0}\left(x\right)$,

(17) $$\nabla_{x}\log{\hat{p}}_{0}\left(x\right)=\frac{\nabla_{x}{\hat{p}}_{0}\left(x\right)}{{\hat{p}}_{0}\left(x\right)}=\frac{\sum_{\ell =1}^{L}c_{\ell }\left(T\right)e^{-\lambda_{\ell }T}\nabla_{x}\varphi_{\ell }\left(x\right)}{\sum_{\ell =1}^{L}c_{\ell }\left(T\right)e^{-\lambda_{\ell }T}\varphi_{\ell }\left(x\right)},$$

where the gradient of the eigenfunction is

(18) $$\nabla_{x}\varphi_{\ell }\left(x\right)=\sum_{m=1}^{M}u_{\ell ,m}\nabla_{x}k\left(x_{m},x\right)=\sum_{m=1}^{M}u_{\ell ,m}\left(-\frac{x-x_{m}}{\zeta^{2}}\right)k\left(x_{m},x\right),$$

using the derivative of the Gaussian kernel $\nabla_{x}k\left(x_{m},x\right)=-\frac{x-x_{m}}{\zeta^{2}}k\left(x_{m},x\right)$.

With the score $\nabla_{x}\log{\hat{p}}_{0}\left(x\right)$, we employ the corrector-only component of the PC sampler from Song et al. [4], specifically annealed Langevin dynamics, to generate samples. Starting from an initial sample $x_{0}^{\left(0\right)}$, we iterate:

(19) $$x_{0}^{\left(j+1\right)}=x_{0}^{\left(j\right)}+\epsilon \nabla_{x}\log{\hat{p}}_{0}\left(x_{0}^{\left(j\right)}\right)+\sqrt{2\epsilon }z_{j},\;j=0,1,\ldots ,J-1,$$

where ϵ>0 is the step size, $z_{j}∼N\left(0,I\right)$ is standard Gaussian noise, and *J* is the number of iterations.

The sampling procedure is summarized in Algorithm 1.
**Algorithm 1** Fast sampling via score-based Langevin dynamics.**Require:** 
Estimated density ${\hat{p}}_{0}\left(x\right)$ from (16), eigenfunctions $\varphi_{\ell }\left(x\right)$, coefficients $c_{\ell }\left(T\right)e^{-\lambda_{\ell }T}$, step size ϵ, number of iterations *J*. 1:Initialize $x_{0}^{\left(0\right)}$. 2:**for **j=0 to J−1 **do** 3:    Compute score $\nabla_{x}\log{\hat{p}}_{0}\left(x_{0}^{\left(j\right)}\right)=\frac{\sum_{\ell =1}^{L}c_{\ell }\left(T\right)e^{-\lambda_{\ell }T}\nabla_{x}\varphi_{\ell }\left(x_{0}^{\left(j\right)}\right)}{\sum_{\ell =1}^{L}c_{\ell }\left(T\right)e^{-\lambda_{\ell }T}\varphi_{\ell }\left(x_{0}^{\left(j\right)}\right)}$ using (17) and (18). 4:    Sample $z_{j}∼N\left(0,I\right)$. 5:    Update $x_{0}^{\left(j+1\right)}=x_{0}^{\left(j\right)}+\epsilon \nabla_{x}\log{\hat{p}}_{0}\left(x_{0}^{\left(j\right)}\right)+\sqrt{2\epsilon }z_{j}$. 6:**end for** 7:**return **$x_{0}^{\left(J-1\right)}$.

Our method significantly speeds up the sampling process by eliminating the predictor step and using only the corrector component of a standard predictor–corrector (PC) sampler. While PC sampling involves $O(N^{2}\cdot M_{\mathrm{nn}})$ nonlinear neural network evaluations (N≈1000, where $M_{\mathrm{nn}}$ denotes the network cost per step), our method performs O(N·L·M) operations consisting entirely of linear vector and matrix computations, where *N* is the number of Langevin iterations, *L* is the number of eigenfunctions, and *M* is the number of reference data points. The proposed approach improves computational efficiency by replacing repeated nonlinear score function evaluations with linear combinations of kernel-based eigenfunctions.

The overall approximation error of the proposed method arises from three sources: (i) operator approximation error due to representing the infinite-dimensional Fokker–Planck operator in a finite-dimensional RKHS subspace constructed from *M* reference data points; (ii) spectral truncation error induced by retaining only the leading *L* eigenmodes of the approximated operator; and (iii) sampling error introduced by finite-step Langevin dynamics. Thus, the total error can be understood as the combination of projection error, spectral truncation error, and discretization error, while a rigorous quantitative bound for the full pipeline is left for future investigation.

**Remark 3.** 
*The quality of generated samples depends on the accuracy of $\nabla_{x}\log{\hat{p}}_{0}\left(x\right)$, which is sensitive to the truncation parameter L and kernel bandwidth ζ. Errors in density estimation may lead to biased scores, potentially degrading sample quality compared to SDE-based methods. Increasing J or tuning ϵ via signal-to-noise ratio optimization [4] can mitigate these effects.*

## 4. Experiments

We conduct experiments to evaluate the proposed fast sampling method, which leverages kernel-based Fokker–Planck eigenanalysis to accelerate score-based generative modeling. Our primary goal is to compare the sampling efficiency and generation quality of our method against the original predictor–corrector (PC) sampling approach from Song et al. [4]. Additionally, we analyze the impact of the number of data points *M* on performance. All experiments are implemented in PyTorch 2.0.1 and run on an NVIDIA RTX 3090Ti GPU.

### 4.1. Comparison with Original Methods

We evaluate our fast sampling method on the CIFAR-10 dataset. We use two score-based models from Song et al. [4]: DDPM++ cont. with VP SDE and NCSN++ cont. with VE SDE, trained with continuous-time objectives (3).

For baselines, we use the PC1000 samplers from Song et al. [4] with 1000 discretization steps. Our method employs a Gaussian kernel *k* with bandwidth ζ=22, selected via cross-validation [18] for constructing the Gram matrices (7). We use M=2000 training data points and select L=5 eigenfunctions based on the dominant eigenvalues of the Fokker–Planck operator. Due to the eigenfunction-based sampling method’s tendency to concentrate samples around high-density regions, resulting in limited diversity within samples generated from a single set of eigenfunctions, we computed 250 different sets of eigendecompositions, each generating 200 images, to produce a total of 50,000 samples. Generation quality is measured by Fréchet Inception Distance (FID) and Inception Scores (ISs) over 50,000 samples and sampling efficiency by time for 1 and 100 images.

For sampling, we use corrector-only Langevin dynamics with step size ϵ=0.5 and J=1000 iterations. Notably, when generating new data via Langevin dynamics, we adopt a smaller bandwidth $\zeta_{\mathrm{sample}}=5<\zeta$ for the kernel in the score computation (17). This adjustment is motivated by the finding that a smaller bandwidth enhances the retention of fine-grained details in the generated images, resulting in better visual quality. A reduced $\zeta_{\mathrm{sample}}$ increases the kernel’s sensitivity to local variations, enabling it to better capture intricate structures within the data distribution. However, this heightened sensitivity can lead to numerical instability in the score computation, particularly when ${\hat{p}}_{0}\left(x\right)$ approaches zero, potentially causing computational errors such as NaN values. To address this, we introduce a noise scale parameter η into the Langevin dynamics update:

$$x_{0}^{\left(j+1\right)}=x_{0}^{\left(j\right)}+\epsilon \nabla_{x}\log{\hat{p}}_{0}\left(x_{0}^{\left(j\right)}\right)+\eta \sqrt{2\epsilon }z_{j}.$$

By setting η=0.2, we scale down the magnitude of the stochastic term, stabilizing the sampling process and preventing numerical overflow while preserving the benefits of a smaller bandwidth.

Furthermore, our experiments revealed that initializing the Langevin dynamics from the standard normal distribution N(0,I), a common practice in score-based generative models, often results in mode collapse when using our eigenfunction-based generation approach. This manifests as generated images converging to nearly identical samples, failing to reflect the diversity of the underlying data distribution. To mitigate this, we initialize the sampling process with solid-color images of varying hues. These initial images are generated by uniformly sampling random RGB values and scaling them to match the dataset’s pixel range. This strategy provides a diverse set of starting points, encouraging the Langevin dynamics to explore distinct regions of the data manifold and effectively alleviating mode collapse.

Table 1 presents the performance of our proposed fast sampling method compared to the original PC1000 sampler on CIFAR-10. Our method aims to achieve significant speedups by replacing iterative SDE solving with linear operations based on Fokker–Planck eigenfunctions.

**Figure 1:**
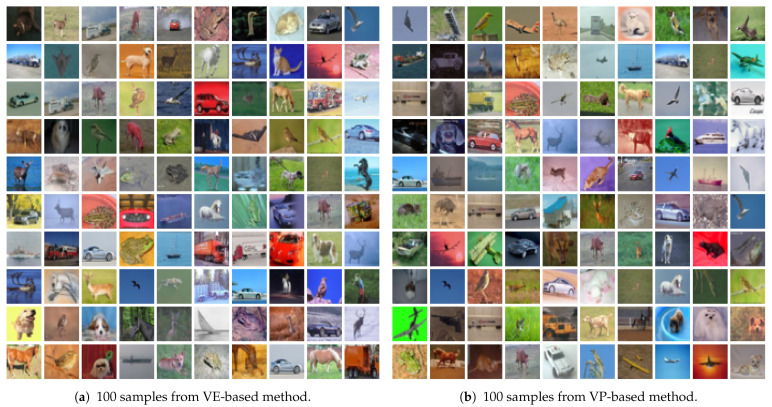
Generated samples on CIFAR-10 using our fast sampling method, arranged in 10 × 10 grids. (**a**) VE-based method. (**b**) VP-based method.

Our proposed fast sampling method, utilizing Fokker–Planck eigenfunctions and corrector-only Langevin dynamics, achieves remarkable efficiency gains on CIFAR-10 compared to the PC1000 samplers from Song et al. [4]. For the VE-based NCSN++ cont. model, single-image sampling time drops from 75.85 s to 0.29 s (261.6× speedup) and 100-image sampling time reduces from 381.32 s to 15.97 s (23.9× speedup). For the VP-based DDPM++ cont. model, sampling times decrease from 35.93 s to 0.30 s (119.8× speedup) for one image and from 176.77 s to 16.08 s (11.0× speedup) for 100 images. However, generation quality declines, with FID scores rising from 2.20 to 42.03 for VE-based and from 2.41 to 43.95 for VP-based methods, indicating reduced sample diversity [20]. Similarly, the IS drops from 9.89 to 8.34 for VE-based and from 9.68 to 8.33 for VP-based methods, reflecting a moderate degradation in sample quality. These results underscore our method’s computational advantages but highlight challenges in maintaining high-fidelity outputs. Figure 1 showcases 100 samples from our VE-based and VP-based methods, illustrating their visual quality. Future work will focus on refining eigenfunction approximations and optimizing kernel parameters to improve FID and IS while preserving efficiency.

### 4.2. Comparison with Efficient Diffusion Solvers

To further contextualize our approach among existing efficient diffusion solvers, we additionally compare it with Denoising Diffusion Implicit Models (DDIM) [5], DPM-Solver [6], and AMED-Solver. These methods represent state-of-the-art approaches designed to accelerate diffusion sampling through improved numerical integration schemes. All evaluations were conducted on the CIFAR-10 dataset under consistent experimental settings with those in Table 1. Table 2 summarizes the quantitative results.

All three solvers achieve strong FID scores (<3.3) while requiring sub-second runtime for generating 100 images, demonstrating impressive efficiency in iterative sampling. In contrast, our proposed operator-theoretic method, reported in Table 1, is fundamentally different in design philosophy: it replaces iterative score evaluations with a closed-form spectral approximation derived from the Fokker–Planck operator. This leads to remarkable speedups of over 100× in single-image generation time (0.29 s versus 35–75 s for PC1000), albeit at the cost of higher FID and slightly reduced Inception Scores.

### 4.3. Ablation Study

#### 4.3.1. Effect of Training Data Size

To investigate the impact of the number of training data points *M* on generation quality and sampling efficiency, we conduct an ablation study using the VE-based NCSN++ cont. model on CIFAR-10. We vary M∈{500,1000,2000,3000,5000}, keeping all other hyperparameters identical to those used in the VE-based method in Section 4.1, including the Gaussian kernel bandwidth, number of eigenfunctions, Langevin iterations, step size, and noise scale. FID is computed over 50,000 samples, and we report sampling times for generating a single image and 100 images to assess computational overhead. Results are shown in Table 3.

Table 3 reveals a non-monotonic relationship between *M* and generation metrics, highlighting a trade-off between sample diversity, quality, and computational cost. Our method generates 50,000 samples using 250 feature groups, each derived from *M* randomly sampled data points. For M=500 to 2000, the IS rises slightly from 8.19 to 8.34, indicating improved sample quality due to more precise density and score function estimation via Gram matrices (7). Conversely, FID increases from 24.58 to 42.03, reflecting reduced sample diversity. This trend stems from the random selection of *M* data points: smaller *M* introduces greater variability across feature groups, enhancing dataset-level diversity, whereas larger *M* concentrates samples around dominant data modes, reducing diversity and worsening FID [20]. Beyond M=2000, IS declines to 7.43 at M=5000, suggesting degraded sample quality due to overfitting in the kernel-based eigendecomposition, which overemphasizes high-density regions and fails to capture the full distribution [12]. These results suggest that moderate *M* values (e.g., 500–1000) offer a favorable balance of diversity, quality, and efficiency, while our choice of M=2000 in Section 4.1 prioritizes quality for practical applications. Future work could explore dynamic *M* selection to optimize this trade-off.

#### 4.3.2. Effect of Number of Dynamic Modes

In this subsection, we examine how the number of retained dynamic modes *L*, i.e., the number of eigenfunctions used in the spectral expansion (16), affects the performance of our generative model. Retaining more modes may provide a richer representation of the underlying density, potentially improving generation quality. However, increasing *L* also introduces greater computational overhead and the risk of amplifying noisy or less informative components of the spectrum.

To isolate the impact of *L*, we fix the number of training data points at M=2000, which was used in Section 4.1. All other hyperparameters remain consistent, including the Gaussian kernel bandwidth, Langevin step size, number of iterations, and noise scale. We evaluate L∈{1,3,5,10,20}, measuring FID, IS, and wall-clock time for generating 1 and 100 images. Results are summarized in Table 4.

From Table 4, we observe that varying the number of retained dynamic modes *L* has a relatively limited effect on generation performance. Across all tested values from L=1 to L=100, both FID and IS remain within a narrow band, with no clear improvement trend as *L* increases. Although the best numerical performance occurs at L=5, the differences across settings are marginal—less than 2 points in FID and about 0.2 in IS—indicating that generation quality is largely robust to the choice of *L*.

This stability suggests that the dominant structural information of the data distribution is already captured by the leading few eigenfunctions. In kernel-based eigendecomposition, it is common for the spectrum to decay rapidly, with most of the meaningful variation encoded in the top modes. Therefore, expanding the spectral basis beyond a small number of components (e.g., L>5) contributes little additional expressive power and may even introduce redundant or noisy modes.

Moreover, sampling time remains nearly constant across all values of *L*, with single-image generation fluctuating between 0.29 and 0.35 s. This confirms that the overall runtime is primarily driven by Langevin dynamics and kernel evaluations, rather than the number of eigenfunctions used.

These results imply that our method is computationally efficient and numerically stable even with a small spectral basis. Using only a few dynamic modes is sufficient for approximating the data density and generating samples of competitive quality. This property not only reduces memory and computational requirements but also simplifies hyperparameter selection in practical deployments.

### 4.4. Initialization-Driven Generation

In this subsection, we investigate whether and to what extent the initial state used in Langevin dynamics influences the output of our kernel-based generative model. While standard score-based diffusion models typically initialize from an isotropic Gaussian distribution, our method permits arbitrary initialization, making it possible to study how structured inputs may affect generation.

**Figure 2:**
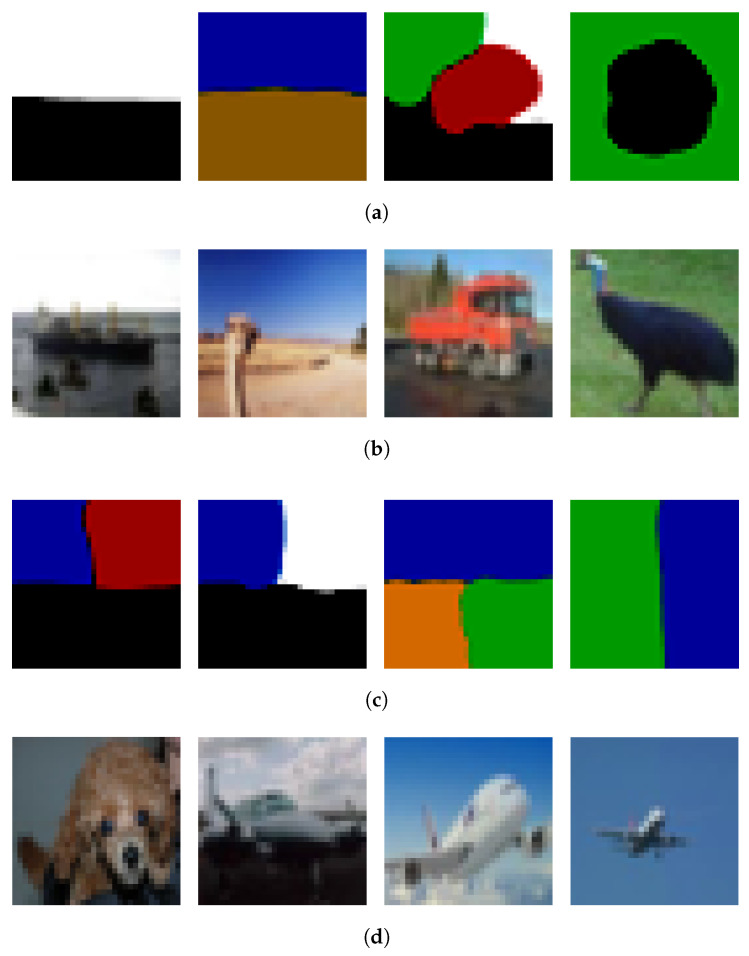
Initialization-driven generation. (**a**) First row: Simple initializations. (**b**) Second row: Generated from simple initializations. (**c**) Third row: Complex initializations. (**d**) Fourth row: Generated from complex initializations. Each column shows an initialization (top) and the corresponding generated sample (bottom). The (top) block uses simple initializations, while the (bottom) block uses complex initializations.

To this end, we replace the standard Gaussian initialization with synthetic images that contain simple patterns, such as colored blocks, edges, or silhouette-like shapes. All other settings remain the same as in Section 4.3.1, including the use of M=5000 training samples. Only the initial sample $x_{0}^{\left(0\right)}$ is modified. Representative outcomes are shown in Figure 2.

We find that the degree to which initialization influences the generated sample is variable and generally unpredictable. In some cases, the initialization appears to guide the output toward a similar color distribution or spatial layout, while in other cases—even with comparable initial patterns—the resulting samples resemble typical outputs from the learned distribution, showing little relation to the input. This inconsistency suggests that while the initialization can have an observable impact, the model lacks robustness in preserving or propagating input structure through the sampling process.

Overall, these findings indicate that the generation process is sensitive to initialization but lacks reliable controllability. The influence of the initial state depends not only on the structure of the input but also on its alignment with high-probability regions under the learned density and the inherent randomness in Langevin dynamics. This highlights a limitation of the current kernel-based framework and motivates future work on incorporating structured priors, conditioning mechanisms, or constraint-aware dynamics to achieve more controllable generation.

## 5. Conclusions

This work introduces a transformative framework for accelerating score-based generative models grounded in SDEs, achieving a significant leap in sampling efficiency while advancing the theoretical underpinnings of diffusion-based generative modeling. By integrating kernel-based Fokker–Planck eigenanalysis within an RKHS, our approach redefines the sampling process, replacing computationally intensive iterative SDE solvers with linear operations derived from the operator’s eigenfunctions. This innovation enables unprecedented speedups, reducing single-image sampling times on CIFAR-10 to approximately 0.29 s, a 100–260× improvement over traditional predictor–corrector methods. The framework’s ability to estimate probability density functions via eigenfunction expansions offers a novel analytical perspective, bridging operator theory from mathematical physics with modern generative modeling. Furthermore, our method’s adaptability to both variance-preserving and variance-exploding SDEs underscores its generality, paving the way for applications across diverse diffusion models, including latent diffusion and flow-based approaches. The proposed fast sampling algorithm, leveraging corrector-only Langevin dynamics with strategic initialization and noise scaling, provides a practical tool for real-time generative tasks, such as interactive image synthesis and on-device generation, thereby broadening the accessibility of high-quality generative models.

Despite these advances, our approach exhibits limitations in sample diversity and quality, as evidenced by elevated FID and reduced IS scores compared to baseline methods. These challenges stem from the sensitivity of eigenfunction approximations to hyperparameters and the potential for overfitting in density estimation with large data subsets. Future work will focus on enhancing density estimation accuracy through adaptive kernel bandwidth selection and exploring hybrid sampling strategies that combine our linear framework with iterative refinements to boost sample fidelity. Additionally, extending the framework to higher-dimensional or multimodal datasets and integrating it with latent diffusion models could further amplify its impact, fostering scalable and efficient generative modeling solutions for real-world applications.

## Figures and Tables

**Table 1:** CIFAR-10 sample time cost and quality.

Model and Method	Time (1 Image, s)	Time (100 Images, s)	FID ↓	IS ↑
DDPM++ cont. (VP, PC1000)	35.93	176.77	2.41	9.68
NCSN++ cont. (VE, PC1000)	75.85	381.32	2.20	9.89
Ours (VP-based)	0.30	16.08	43.95	8.33
Ours (VE-based)	0.29	15.97	42.03	8.34

**Table 2:** Comparison of recent efficient diffusion solvers on CIFAR-10.

Method	FID ↓	Time (1 Image, s)	Time (100 Images, s)
DDIM (50 steps)	3.26	0.96	5.78
DPM-Solver (20 steps)	3.15	0.51	0.95
AMED-Solver (20 steps)	2.95	0.42	1.49

**Table 3:** Ablation study on the impact of *M* on VE-based sampling on CIFAR-10.

*M*	FID ↓	IS ↑	Time (1 Image, s)	Time (100 Images, s)
500	24.58	8.19	0.29	4.16
1000	33.91	8.33	0.29	8.22
2000	42.03	8.34	0.29	15.97
3000	52.29	7.76	0.40	24.12
5000	65.97	7.43	0.55	40.21

**Table 4:** Ablation study on the effect of *L* on VE-based sampling on CIFAR-10.

*L*	FID ↓	IS ↑	Time (1 Image, s)	Time (100 Images, s)
1	43.73	8.20	0.31	15.92
3	44.08	8.13	0.31	16.09
5	42.03	8.34	0.29	15.97
10	44.43	8.18	0.30	16.04
20	44.32	8.12	0.33	16.07
50	44.31	8.12	0.31	17.15
100	43.99	8.10	0.35	16.14

## Data Availability

No new data were created or analyzed in this study. Data sharing is not applicable to this article.
